# Selective bacterial degradation of the extracellular matrix attaching the gingiva to the tooth

**DOI:** 10.1111/eos.12623

**Published:** 2019-06-22

**Authors:** Aurélien Fouillen, Daniel Grenier, Jean Barbeau, Christian Baron, Pierre Moffatt, Antonio Nanci

**Affiliations:** ^1^ Laboratory for the Study of Calcified Tissues and Biomaterials Université de Montréal Montréal QC Canada; ^2^ Department of Stomatology Faculty of Dental Medicine Université de Montréal Montréal QC Canada; ^3^ Department of Biochemistry and Molecular Medicine Faculty of Medicine Université de Montréal Montréal QC Canada; ^4^ Oral Ecology Research Group Faculty of Dentistry Université Laval Quebec City QC Canada; ^5^ Shriners Hospitals for Children Montréal QC Canada

**Keywords:** junctional epithelium, periodontal diseases, *Porphyromonas gingivalis*, specialized basal lamina, supramolecular network

## Abstract

The junctional epithelium (JE) is a specialized portion of the gingiva that seals off the tooth‐supporting tissues from the oral environment. This relationship is achieved via a unique adhesive extracellular matrix that is, in fact, a specialized basal lamina (sBL). Three unique proteins – amelotin (AMTN), odontogenic ameloblast‐associated (ODAM), and secretory calcium‐binding phosphoprotein proline‐glutamine rich 1 (SCPPPQ1) – together with laminin‐332 structure the supramolecular organization of this sBL and determine its adhesive capacity. Despite the constant challenge of the JE by the oral microbiome, little is known of the susceptibility of the sBL to bacterial degradation. Assays with trypsin‐like proteases, as well as incubation with *Porphyromonas gingivalis, Prevotella intermedia*, and *Treponema denticola*, revealed that all constituents, except SCPPPQ1, were rapidly degraded. *Porphyromonas gingivalis* was also shown to alter the supramolecular network of reconstituted and native sBLs. These results provide evidence that proteolytic enzymes and selected gram‐negative periodontopathogenic bacteria can attack this adhesive extracellular matrix, intimating that its degradation could contribute to progression of periodontal diseases.

The tooth is the only structure of the body that breaches the epithelial barrier. Its interface with the gingiva surrounding the tooth is therefore of critical importance in periodontal pathogenesis 1, 2, as well as for preventing systemic dissemination of bacteria 3. The so‐called dentogingival junction is composed of a sulcular epithelium (SE) and a junctional epithelium (JE). The SE extends above the JE and is separated from the tooth by a natural space that creates an ecological niche favorable for microbial colonization. The JE below adheres to the mineralized tooth surface to prevent the spread of oral bacteria from the sulcus into the subjacent tooth‐supporting tissues and their destruction when periodontal diseases set in.

The JE cells form a specialized basal lamina (sBL) to achieve a strategic adhesive relationship with the tooth surface. This extracellular matrix is enriched in laminin‐332 (Lm332) and contains no type IV and VII collagens, setting it apart from typical basal laminae that bind to connective tissues 4. The JE additionally secretes three unique epithelial proteins: amelotin (AMTN); odontogenic ameloblast‐associated (ODAM); and secretory calcium‐binding phosphoprotein proline‐glutamine rich 1 (SCPPPQ1). Our current state of knowledge suggests that the supramolecular network of the sBL comprises only these four epithelial components. Our group has established, by immunogold labeling, that AMTN, ODAM, and SCPPPQ1 conspicuously localize to the sBL 5, 6. We proposed that they contribute to structuring the supramolecular organization of the sBL that determines its adhesive capacity 5, 7.

Periodontitis is a multifactorial disorder that leads to destruction of tooth‐supporting tissue, ultimately resulting in irreversible damage and tooth loss 8. It is well recognized that microorganisms constitute the primary etiologic agents of periodontitis 9, 10, 11. Human subgingival plaque harbors more than 700 species of bacteria. Among these, only a minority is virulent and leads to periodontitis when the equilibrium of the oral microbiome is compromised 8. Among others, *Porphyromonas gingivalis* and *Treponema denticola* are considered as late colonizers and are strongly associated with active periodontitis lesions. They are usually found in the presence of bridging colonizer species, such as *Prevotella intermedia*,* Aggregatibacter actinomycetemcomitans*, and *Fusobacterium nucleatum*, whose roles are to stimulate and facilitate the aggregation of the late colonizers 12. Some oral bacteria secrete a variety of virulence factors 13. For instance, *P. gingivalis* – one of the most studied oral bacterial species – produces a group of enzymes named gingipains that are usually associated with connective tissue destruction and that are involved in colonization as well as in perturbation of host defense 14. It has also recently been proposed that multispecies bacterial biofilms release a factor that affects the cellular integrity and protective role of the JE against periodontitis 15.

The JE at the bottom of the sulcus is susceptible to bacteria that accumulate there and could attack the JE and perturb its functional and structural integrity 1, 16. Such perturbation creates a space, referred to as a periodontal pocket, that is of particular relevance as bacteria can now directly deliver their toxins along a larger surface. This expanded activity prevents reattachment and aggravates cellular dysfunction, extending damage beyond the JE to the tooth‐supporting tissues 11. As such, transformation of the JE into a pocket epithelium is considered as a determinant trait in the development of periodontitis 2.

Despite the importance of periodontal pockets, the mechanisms leading to their initiation are still obscure 17. Disruption of the adhesive interface would inexorably favor JE detachment and periodontal pocket formation. Yet, little is known regarding the susceptibility of the adhesive sBL to degradation by bacteria to which it is continuously exposed 17. Our objective was therefore to determine whether bacteria from the oral microbiome can degrade the individual components of the sBL, thereby affecting its supramolecular organization and functionality. Individual proteins constituting the sBL were purified and exposed to selected periodontopathogenic bacteria and to proteases. We also evaluated bacterial activity ex vivo on a reconstituted sBL and on the native sBL itself. All components of the sBL, except SCPPPQ1, were found to be susceptible to some periodontopathogenic bacteria. Both reconstituted and native sBLs were also degraded. These results demonstrate, for the first time, that the sBL can be the target of degradation by bacteria known to play a major role in periodontal diseases.

## Material and methods

All animal procedures were approved by the Comité de Déontologie de l'Expérimentation sur les Animaux of Université de Montréal, and all methods were performed in accordance with their guidelines and regulations.

### Cloning procedures

Truncated versions of *ODAM, AMTN*, and *SCPPPQ1* (lacking regions encoding the predicted N‐terminal signal sequence) were PCR‐amplified from human cDNA sequences using primers as previously described 5. The PCR products were cloned into the vector, pHT, for purification studies 5. The recombinant pHT plasmids allow production of recombinant proteins with an in‐frame N‐terminal hexahistidyl‐tag (His‐tag) and a TEV protease cleavage site. *Escherichia coli* strain XL‐1 Blue was used as host for cloning 5.

### Protein overexpression and purification

BL21(DE3)‐star cells containing either pHT‐*hAMTN* or pHT‐*hODAM* 5 were grown at 37°C with shaking (at 250 r.p.m.) to an optical density at 660 nm (OD_660_) of around 0.6, and protein expression was induced with 0.1 mM isopropyl‐*β*‐d‐thiogalactoside (IPTG) for 5 h at 30°C with shaking (at 250 r.p.m.). Bacterial cells were harvested, suspended in equilibration buffer (50 mM Na_2_HPO_4_, 150 mM NaCl, 10 mM imidazole, pH 7) at 4°C, and sonicated six times for 15 s (with incubation on ice for an intervening period of 15 s between each sonication procedure). Lysates were centrifuged at 13,400 ***g***, and the 6His‐tagged protein in the supernatant was bound on nickel‐nitriloacetic acid (Ni‐NTA)‐agarose affinity resin (Qiagen, Valencia, CA, USA) at room temperature. After washing the resin with 10 volumes of binding buffer (50 mM Na_2_HPO_4_, 300 mM NaCl, 20 mM imidazole, pH 7), proteins were eluted with elution buffer (50 mM Na_2_HPO_4_, 300 mM NaCl, 300 mM imidazole, pH 7). Collected fractions were assessed for protein content using the Bradford assay 18 and analyzed by SDS‐PAGE and Coomassie blue staining. Fractions containing the highest levels of proteins were then dialyzed into TEV buffer (25 mM Na_2_HPO_4_, 125 mM NaCl, 5 mM DTT, pH 7.4) and the N‐terminal His‐tag of proteins in these fractions was cleaved using His‐tagged TEV protease (Sigma‐Aldrich, St Louis, MO, USA) in a 1:70 ratio (TEV protease: protein; w/w) overnight at room temperature (20 to 25°C). Following cleavage, the solution was applied to an Ni‐NTA‐agarose affinity resin and the flow‐through containing cleaved protein was collected. Proteins were dialyzed into 50 mM Na_2_HPO_4_ (pH 7.2) and stored at 4°C. Finally, proteins were purified by size‐exclusion chromatography using a Superose 6 GL 10/300 gel filtration column for ODAM or a S200 gel filtration column for AMTN (GE Healthcare, Chicago, IL, USA). Columns were pre‐equilibrated with 50 mM Na_2_HPO_4_ (pH 7.4). Fractions were evaluated for purity by SDS‐PAGE. Secretory calcium‐binding phosphoprotein proline‐glutamine rich 1 was expressed in pHT‐*hSCPPPQ1* and grown and purified in the same conditions as ODAM and AMTN but under denaturing conditions in which buffers contained 8 M urea. Purified Lm332 was commercially obtained (EUV101; KeraFast, Boston, MA, USA).

### Prediction of cleavage sites

The tool ‘Peptide cutter’ (ExPASy; www.expasy.org) was used to predict potential substrate cleavage sites cleaved by chosen proteases in a given protein sequence. We have evaluated how eight proteases from the Proti‐Ace and Proti‐Ace 2 kits (Hampton Research, Aliso Viejo, CA, USA) were able to cleave the proteins from the sBL. The proteases used for the assay are listed in Table S3.

### Enzymology assay

Twenty micrograms of purified proteins in their final buffer were added to 2.2 *μ*g of eight proteases in 10 mM HEPES (pH 7.5), 500 mM NaCl, from the Proti‐Ace and Proti‐Ace 2 kits (Hampton Research), and incubated at 37°C for 1 h. The resulting products were then analyzed by SDS‐PAGE. As a control, we used 20 *μ*g of purified proteins in 20 *μ*l of enzyme buffer (10 mM HEPES (pH 7.5), 500 mM NaCl).

### Bacterial culture

*Porphyromonas gingivalis* ATCC 33277, *F. nucleatum* ATCC 25586, *P. intermedia* ATCC 25611, and *A. actinomycetemcomitans* ATCC 29522 were grown anaerobically for 24 h (80% N_2_, 10% CO_2_, 10% H_2_) at 37°C in Todd‐Hewitt broth (THB; Becton Dickinson, Mississauga, ON, Canada) supplemented with 0.001% hemin and 0.0001% vitamin K. *Treponema denticola* ATCC 35404 was grown anaerobically for 24 h in liquid medium containing 12.5 mg ml^–1^ of brain heart infusion, 10 mg ml^–1^of trypticase, 2.5 mg ml^–1^ of yeast extract, 0.5 mg ml^–1^ of sodium thioglycolate, 1 mg ml^–1^ of l‐cysteine, 0.25 mg ml^–1^ of l‐asparagine, 2 mg ml^–1^ of glucose, 6 *μ*g ml^–1^ of thiamine pyrophosphate, 2 mg ml^–1^ of sodium bicarbonate, 2% rabbit serum, and 0.2% volatile fatty acids 19. The volatile fatty acids solution consisted of 0.5 ml each of isobutyric, dl‐2‐methylbutyric, isovaleric, and valeric acid dissolved in 100 ml of 0.1 M KOH.

### Tissue preparation

Six Wistar rats of approximately 150 g were anesthetized with 20% chloral hydrate solution (0.4 mg g^−1^ of body weight) (Fisher Scientific, Whitby, ON, Canada) and sacrificed by perfusion through the left ventricle with Ringer's lactate (Abbott Laboratories, Montreal, QC, Canada) for 30 s, followed by a fixative solution consisting of periodate‐lysine‐paraformaldehyde (PLP) in 0.0375 M phosphate buffer (PB) (pH 7.2) for 15 min at room temperature. Periodate‐lysine‐paraformaldehyde is well recognized as a fixative solution that lightly crosslinks samples and thus maximizes the efficiency of the enzymes. Mandibles and maxillae were dissected, and specimens were immersed in the same fixative solution for 15 min at room temperature. The samples were rinsed in 0.0375 M PB (pH 7.2) overnight at 4°C, decalcified with 4.13% disodium ethylenediamine tetra‐acetic acid (EDTA; Fisher Scientific, Hampton, NY, USA) and 0.01% glutaraldehyde for 15 d, then washed for 24 h in 0.0375 M PB (pH 7.2). The decalcified hemimandibles were used to prepare enamel organ caps and the maxillae JE caps.

### Preparation of enamel organ and JE caps

To examine the effect of bacteria on the native sBL and in the absence of any inflammatory components, we prepared maturation‐stage enamel organ caps 20. Segments of incisors containing the maturation‐stage enamel were prepared from the decalcified hemimandibles by first of all making a cut from mesial to the lateral side using a molar reference line 21. Enamel caps were then released by making lateral cuts at their margins, as previously described 20. The caps were then washed extensively in 0.1 M PBS and kept at 4°C until incubation of the whole enamel caps in 1 ml of a suspension of bacteria. It should be pointed out here that the JE derives in part from the enamel organ and that the sBL, which attaches the enamel organ to maturing enamel, is similar in composition to the one attaching the JE to the tooth surface 5, 7, 22, 23, 24, 25. These caps were chosen because their geometry facilitates incubation with suspensions of bacteria and are therefore more suited for large‐scale analysis than JE caps. However, to validate the results obtained with enamel organ caps, some JE caps were also prepared from decalcified maxillary molars by cutting the gingiva circumferentially along the base of the anatomical crown (where the JE normally ends) 23. These other caps were processed and incubated as above.

### Incubation of bacteria with purified proteins

Approximately 20 *μ*M of each of the purified proteins was exposed, in a test tube, to 800 *μ*l of a suspension of bacteria at an OD_660_ of 1 [equivalent to 10^9^ colony‐forming units (CFUs) ml^−1^ 26] [multiplicity of infection (MOI) ≈ 1.2 × 10^6^ molecules per bacteria] for 2 h at 37°C. Such a concentration of *P. gingivalis* was previously used to induce experimental periodontitis in mice 27. Forty microlitres was sampled every 15 min for western blot analysis using antibodies for rat ODAM (1:5,000), rat AMTN (1:2,000), or rat SCPPPQ1 (1:2,000). Protein alone in the bacterial medium was used as a control. The same procedure was applied in the presence of a cocktail of protease inhibitor (Sigma‐Aldrich). The number of bacteria in the suspension at the start and end of the assay was evaluated using field‐emission scanning electron microscopy (FE‐SEM). As a negative control, proteins were incubated with *P. gingivalis* fixed for 30 min with 2.5% glutaraldehyde (Electron Microscopy Sciences, Hatfield, PA, USA) in PB, pH 7.2, followed by three washes also in PB. Western blot gels were acquired using a Bio‐Rad ChemiDoc imager and the software image lab (Bio‐Rad, Hercules, CA, USA). Exposure times for the acquisition were between 1 and 2 s.

### Bacterial incubation with enamel organ and JE caps

Enamel organ and some JE caps were exposed to 1 ml of a suspension of *P. gingivalis* at an OD_660_ of 1 (equivalent to 10^9 ^CFUs ml^−1^) for 2 and 6 h at 37°C followed by fixation and processing for FE‐SEM as described below. As a control, *A. actinomycetemcomitans* was used under the same experimental conditions and also observed by FE‐SEM.

### Top‐down liquid chromatography/tandem mass spectrometry

Before and after incubation with *P. gingivalis*, samples were diluted in 25% acetonitrile (ACN)/0.3% trifluoroacetic acid and loaded onto a 50 × 4.6 mm PLRP‐S 300A column (Agilent Technologies, Santa Clara, CA, USA) connected to an Accela pump (Thermo Scientific, Waltham, MA, USA) and an RTC autosampler (Pal Systems, Zwingen, Switzerland). The buffers used for chromatography were 0.1% formic acid (buffer A) and 100% ACN/0.1% formic acid (buffer B). Proteins and peptides were eluted with a two‐slope gradient at a flow rate of 120 ml min^−1^. Solvent B first increased from 20% to 40% in 70 min and then from 40% to 70% in 5 min. The HPLC system was coupled to an Orbitrap Fusion mass spectrometer (Thermo Scientific) through an electrospray ion source. The spray and S‐lens voltages were set to 3.6 kV and 50 V, respectively. Capillary temperature was set to 225°C. Full‐scan mass spectromentry (MS) survey spectra [mass‐to‐charge ratio (*m*/*z*): 500–1,700] in profile mode were acquired in the Orbitrap with a resolution of 120,000 with a target value at 5e5. The three most intense protein/peptide ions were fragmented in the HCD collision cell and analyzed in the Orbitrap with a target value at 5e5 and a normalized collision energy at 36 V. Target ions selected for fragmentation were dynamically excluded for 25 s.

For data processing, protein database searching was performed using ProSightPC 4.0.2.1 (Thermo Scientific) against the protein sequences of interest. The mass tolerances for precursor and fragment ions were set to 1,000 Da and 20 ppm, respectively. The minimum number of matching fragments was set to 4, and the only modification used was acetylation of proteins N‐terminus. The number of matching fragments were quantified and classified according to molecular weight to evaluate the effect of incubation with *P. gingivalis* on each protein.

### Atomic force microscopy

Atomic force microscopy (AFM) imaging was performed using a JEOL JSPM‐5200 Scanning Probe Microscope (JEOL, Tokyo, Japan). A 10 μl drop of a sample solution containing a mix of AMTN, ODAM, SCPPPQ1, and Lm332 (each protein at 50 nM) was incubated for 5 min on a highly ordered pyrolytic graphic (HOPG) substrate (MicroMasch, Watsonville, CA, USA) to re‐create the reconstituted sBL 5. Then, the HOPG surface was rinsed three times with 50 ml of distilled water and air‐dried. Some samples were incubated with* P. gingivalis* or *A. actinomycetemcomitans* for 1 min or 2 h before rinsing and air‐drying steps. All imaging was observed under dry conditions and was carried out using the tapping mode at room temperature. The cantilevers used (HQ‐NSC14; MicroMasch) had a spring constant of 5.7 N m^−1^. The scan speed was 0.5 Hz and scan size was 1.5 mm. The samples were visualized as topographic images and three‐dimensional (3D) images at a resolution of 512x512 pixels using Gwyddion (gwyddion.net).

### Scanning electron microscopy

Following incubation with bacteria, tissues and bacterial samples were fixed for 1 h at 4°C in 2.5% glutaraldehyde and subsequently rinsed three times with 0.1 M PB, pH 7.3, and incubated for 30 min in 1% osmium tetroxide at room temperature. The tissues were dehydrated through an ethanol series (once in 30%, 50%, 70%, 90%, and 95% ethanol and twice in 100% ethanol) then dried using a Critical Point Drier CPD300 (Leica Biosystems, Concord, ON, Canada). Routine characterization of the samples was carried out using a JEOL JSM‐7400F (JEOL) FE‐SEM operated at 1.5 kV. An FE‐SEM Regulus 8230 (Hitachi, Tokyo, Japan), operated at 0.8 kV, was used for higher resolution images to observe the effects of bacteria on the sBL.

## Results

### In‐vitro susceptibility of sBL proteins to oral bacteria

**Figure 1:**
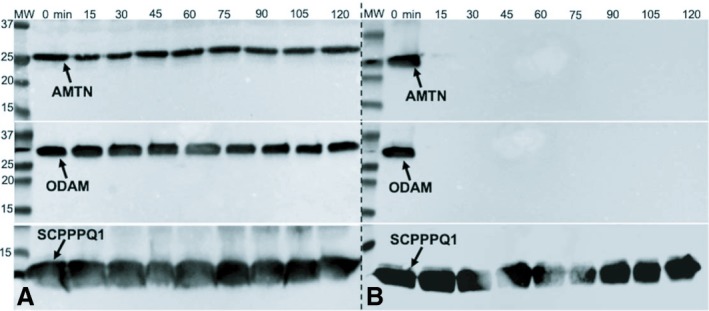
Representative degradation profiles of amelotin (AMTN), odontogenic ameloblast‐associated (ODAM), and secretory calcium‐binding phosphoprotein proline‐glutamine rich 1 (SCPPPQ1) by periodontopathogenic bacteria. Western blot analysis following in‐vitro exposure to (A) *Aggregatibacter actinomycetemcomitans* and (B) *Porphyromonas gingivalis*. No degradation is observed with *A. actinomycetemcomitans*, whereas *P. gingivalis* degrades AMTN and ODAM in under 15 min, but SCPPPQ1 is not affected. Membranes were exposed for 1–2 s. MW in kDa.

Recombinant sBL proteins were incubated separately, for 2 h, with single species of oral bacteria. Western immunoblotting analysis revealed no degradation of protein in the presence of *F. nucleatum* (data not shown) or *A. actinomycetemcomitans* (Fig. 1A). By contrast, incubation of single proteins with *P. gingivalis*,* T. denticola*, or *P. intermedia* (Figure S1) resulted in complete degradation of AMTN and ODAM, but not of SCPPPQ1 (Fig. 1B). SDS‐PAGE and western blot analysis of AMTN and ODAM incubated for shorter periods with *P. gingivalis* revealed almost instantaneous and complete degradation of both proteins (Figure S2). The presence of a cocktail of protease inhibitors decreased the extent of degradation of AMTN, resulting in a persistent band at around 20 kDa (Figure S3A). Under this same condition, degradation of ODAM was still complete (Figure S3B). However, inactivation of *P. gingivalis* by fixation with glutaraldehyde prevented the degradation of both AMTN and ODAM (Figure S3C,D). Laminin‐332 was also completely degraded as early as 5 min following incubation with *P. gingivalis* (Figure S4).

**Figure 2:**
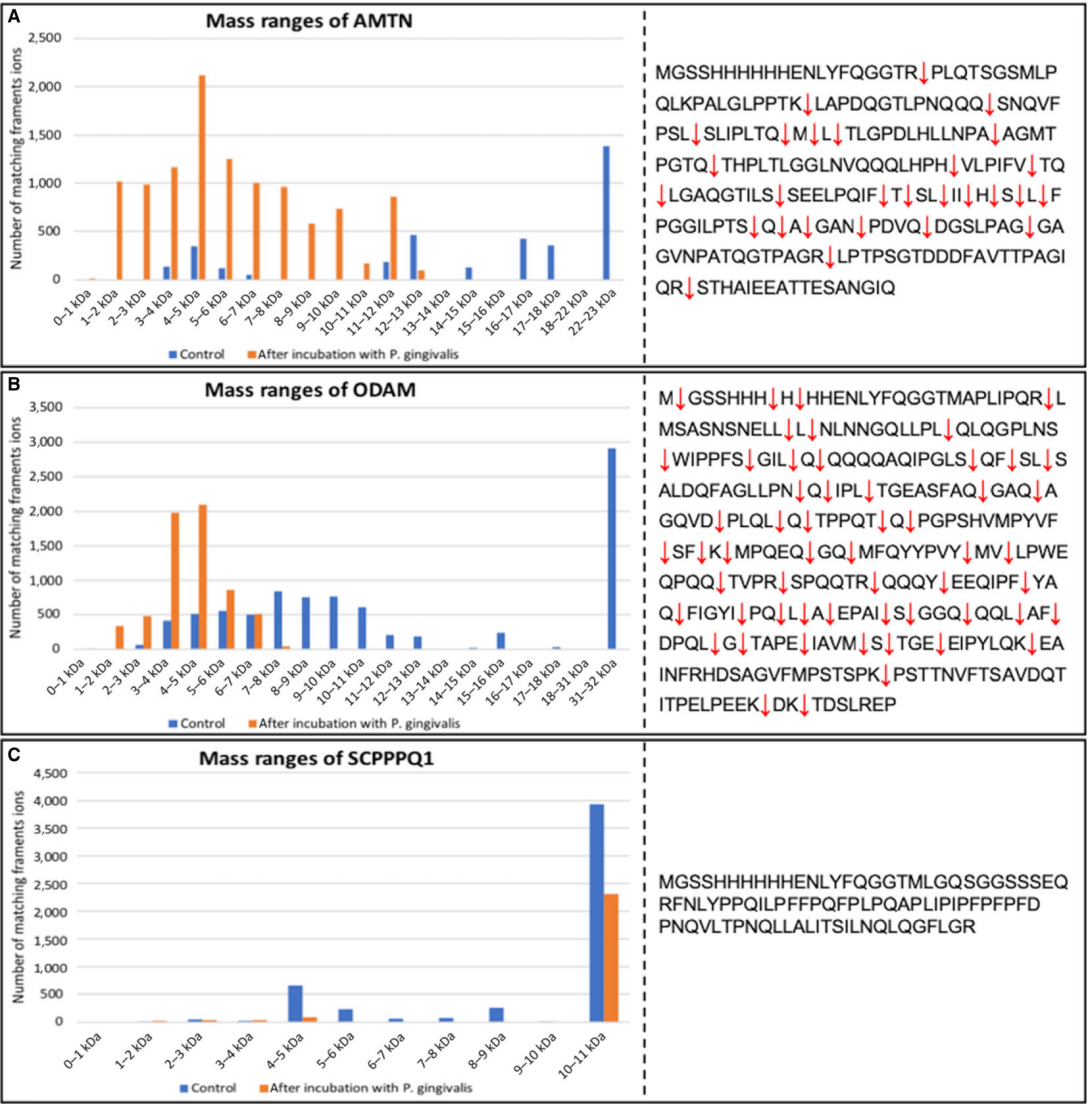
Top‐down mass spectrometry analysis of the cleavage products following in vitro incubation of proteins with *Porphyromonas gingivalis*. (A) Amelotin (AMTN), (B) odontogenic ameloblast‐associated (ODAM), and (C) secretory calcium‐binding phosphoprotein proline‐glutamine rich 1 (SCPPPQ1), before (blue) and after (orange) incubation with *Porphyromonas gingivalis* confirms the degradation of AMTN and ODAM and the non‐degradation of SCPPPQ1 observed by western blot analysis (Fig. 1 and S2). The right panels highlight the cleavage sites (red arrows) for the proteins.

To confirm degradation of the proteins and to determine their cleavage sites, we performed liquid chromatography/tandem mass spectrometry (LC/MS‐MS) using the top‐down assay after incubation of the sBL proteins with *P. gingivalis* (Fig. 2). This species of bacterium extensively cleaved AMTN into multiple fragments of 1–14 kDa and ODAM into multiple fragments of 1–9 kDa. The predominant size of fragments for both proteins was 2–5 kDa (Tables S1 and S2). Secretory calcium‐binding phosphoprotein proline‐glutamine rich 1 was not degraded after incubation with *P. gingivalis*, confirming the above SDS‐PAGE and western blot observations. Under the control condition, at time 0, and when no bacteria were added to the samples during a 2‐h time period, no degradation was observed (Figs 1, 2, S1–S4).

**Figure 3:**
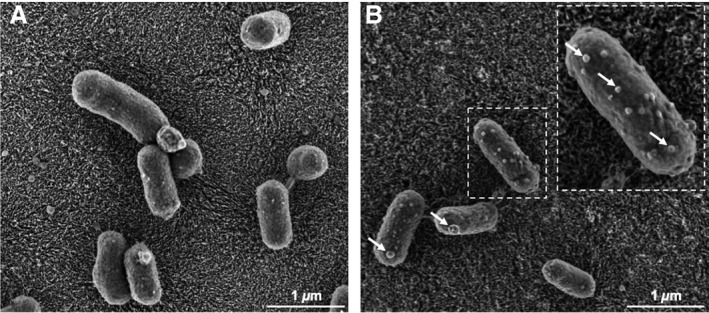
Scanning electron microscopy of *Porphyromonas gingivalis* after 2 h of exposure to (A) buffer alone as the control and (B) buffer containing secretory calcium‐binding phosphoprotein proline‐glutamine rich 1 (SCPPPQ1). Following exposure to the protein, numerous outer membrane vesicles (OMVs) (arrows) appear on the bacterial surface.

At time 0 and 2 h, bacteria were observed by FE‐SEM to evaluate whether degradation of the proteins induced any alteration in their general appearance. A noticeable change related to outer membrane vesicles (OMVs). Based on qualitative observations of multiple samples, bacteria exposed to AMTN, ODAM, or SCPPPQ1 before being placed on a support for FE‐SEM generally exhibited more OMVs on the support, compared with bacteria incubated for the same period of time but in buffer only (Figure S5). *Porphyromonas gingivalis* incubated with SCPPPQ1 also tended to show a larger number of OMVs bulging out from their membrane (Fig. 3).

### Enzymatic degradation of sBL proteins

**Figure 4:**
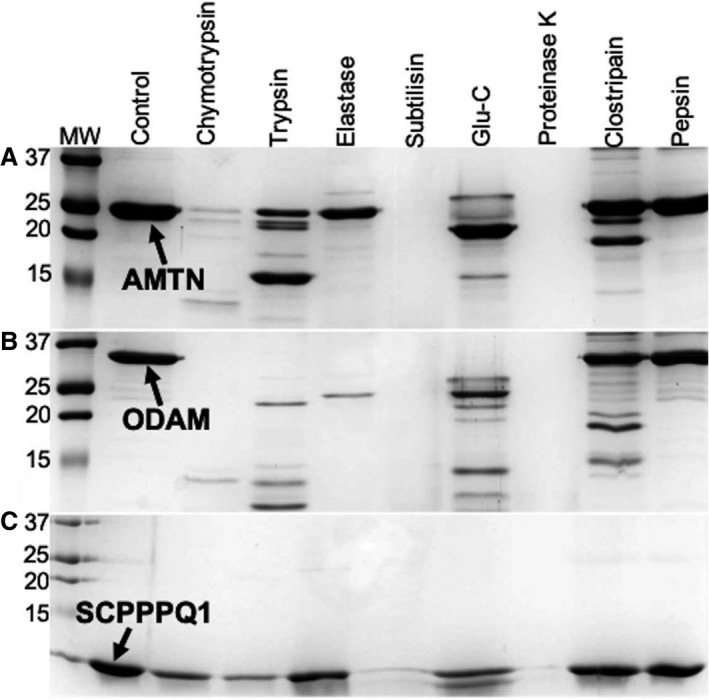
SDS‐PAGE analysis of the in‐vitro digestion of (A) amelotin (AMTN), (B) odontogenic ameloblast‐associated (ODAM), and (C) secretory calcium‐binding phosphoprotein proline‐glutamine rich 1 (SCPPPQ1) with various enzymes reveals that the proteins are differentially susceptible to the various protease families tested. MW in kDa.

To evaluate the classes of proteases that may be involved in degradation of the sBL constituents, first of all an in‐silico analysis was performed using ‘Peptide cutter’ 28. The proteases chosen and their potential number of cleavage sites in each protein are listed in Table S3. The results showed that AMTN, ODAM, and SCPPPQ1 appear to be sensitive, at different levels, to all the enzymes. Next, the proteins were incubated in vitro with each protease to determine whether they are susceptible to cleavage (Fig. 4). The results showed that the proteins are degraded more extensively by enzymes from the serine‐protease family than by cysteine or aspartic proteases. Subtilisin and proteinase K appeared to digest the sBL proteins most efficiently. Trypsin‐like proteases, which are produced by a number of periodontopathogenic bacteria 14, degraded AMTN and ODAM, but not SCPPPQ1. Few cleavage sites are predicted for Lys‐gingipain and Arg‐gingipain individually, but together there was an increase in predicted cleavage sites, except for SCPPPQ1 (Table S3). Mass spectrometry confirmed cleavages at these sites for AMTN and ODAM and none for SCPPPQ1 (Fig. 2). As predicted from in‐silico analysis (Table S3), the endoproteinase, Glu‐C, poorly cleaved the sBL proteins. Finally, the elastase proteases, which target several potential cleavage sites in AMTN, ODAM, and SCPPPQ1, only cleaved ODAM completely.

### Degradation of the supramolecular network of the sBL

**Figure 5:**
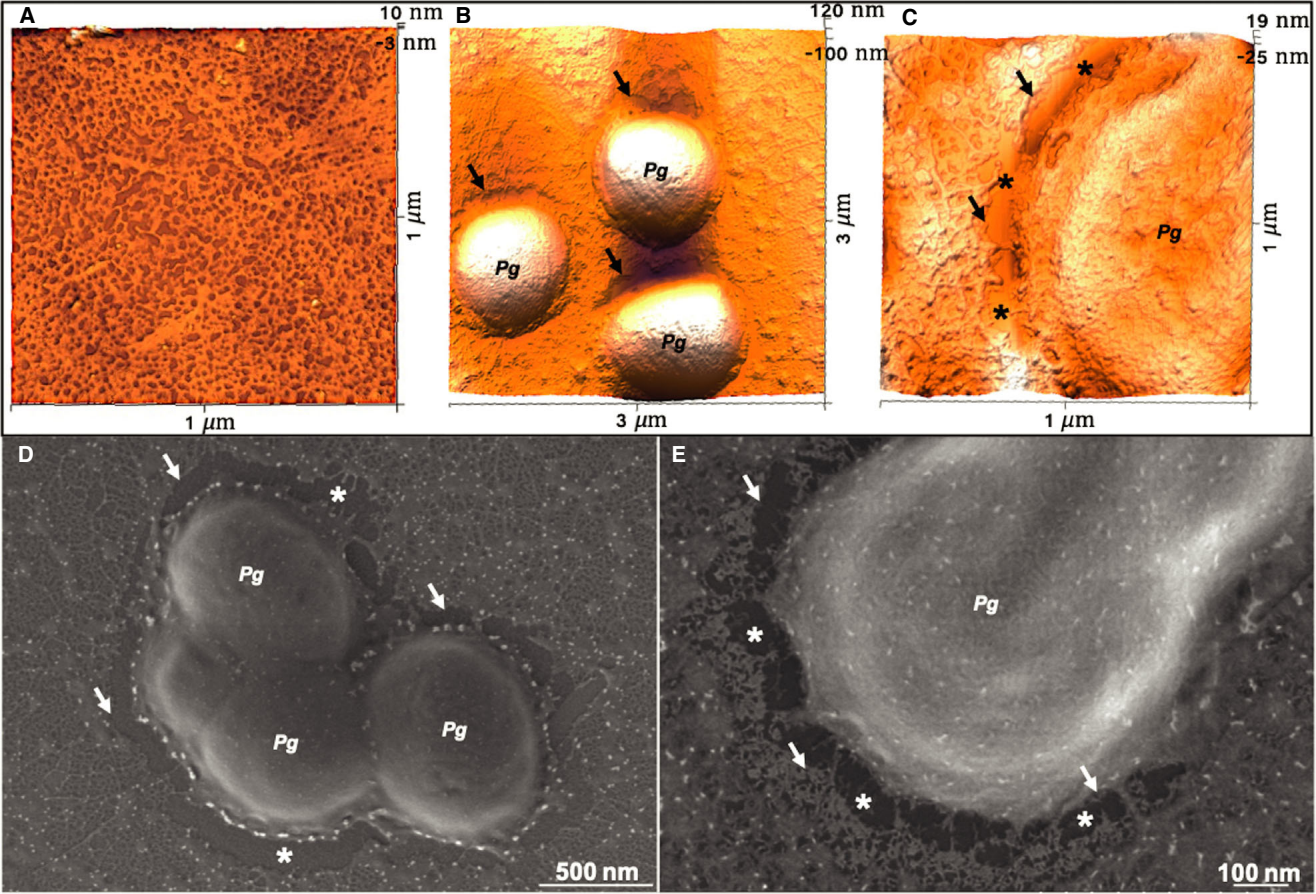
Atomic force microscopy (AFM) observation of degradation of reconstituted specialized basal lamina (sBL) by *Porphyromonas gingivalis*. (A) The supramolecular network formed by amelotin (AMTN), odontogenic ameloblast‐associated (ODAM), secretory calcium‐binding phosphoprotein proline‐glutamine rich 1 (SCPPPQ1), and laminin‐332 (Lm332), and (B, C) its local alteration (arrows) by *P. gingivalis* (*Pg*) *to* create a peribacterial region of destruction. (D, E) Scanning electron microscopy of the AFM preparations confirms the peripheral destruction and additionally shows the presence of residual filamentous material (*) in the affected region.

As we previously reported, mixing of AMTN, ODAM, and SCPPPQ1 results in the formation of a supramolecular network similar to that described for typical basal laminae 5. Sequential application of the four known components of sBL to HOPG substrate for AFM resulted in formation of a nanoporous molecular network. The nanopores had an average diameter of 66.72 nm^2^ when AMTN, ODAM, and SCPPPQ1 were mixed, and of 36.05 nm^2^ when Lm332 was also added (data not shown). To observe the impact of oral bacteria on this ‘reconstituted’ sBL network, we exposed it to *P. gingivalis* and observed the results by AFM (Fig. 5A–C) and FE‐SEM (Fig. 5D, E). There was destruction of the supramolecular network creating a peripheral space around the bacteria [≈10 nm deep, Figs 5 (arrows) and S6], indicating that bacterial products degraded, at least in part, the ‘reconstituted’ sBL network. Exposure of the reconstituted sBL to *A. actinomycetemcomitans* did not produce an area of destruction around the bacteria (Figure S7B–D), which is consistent with the results of the in vitro assay.

### Degradation of the native sBL

**Figure 6:**
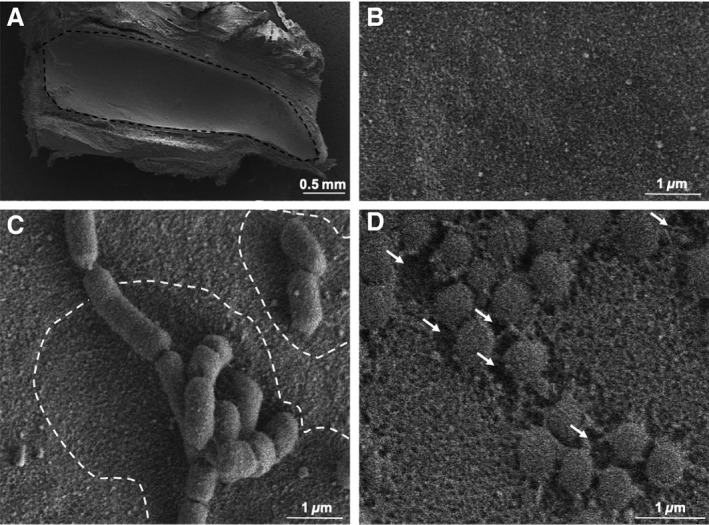
Scanning electron microscopy observations of the native specialized basal lamina (sBL) incubated with *Porphyromonas gingivalis*. (A) Overview (dashed area) and (B) higher‐magnification view of the exposed sBL on an enamel organ cap. Following exposure of the sBL to *P. gingivalis* for 2 h, (C) zones of alteration can be seen around the bacteria (white dashed lines); after 6 h (D) the peribacterial zones appear cavitated (arrow).

**Figure 7:**
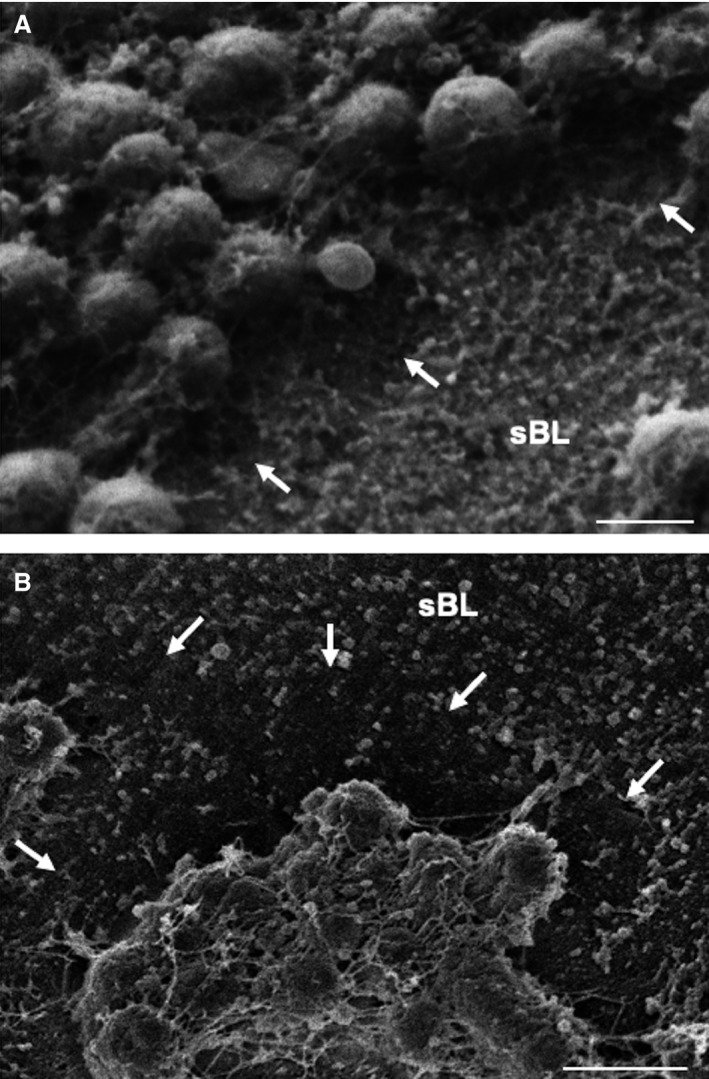
Scanning electron microscopy of junctional epithelium caps exposed to *Porphyromonas gingivalis* (A, B) for 6 h. Similarly to the exposure of enamel organ caps (Fig. 6), there are peribacterial alterations of the native specialized basal lamina (sBL) (arrows). Scale bar = 1 μm.

Finally, to determine the effect of *P. gingivalis* on the native sBL itself, in the absence of any inflammatory contribution, we exposed microdissected enamel organ caps (Fig. 6) and JE samples (Fig. 7) 20, 23 to *P. gingivalis. *After 2 h of exposure, FE‐SEM revealed the presence of a shallow depression around the bacteria (Fig. 6C) and, after 6 h, the affected peri‐bacterial area became deeper (Fig. 6D), suggesting active degradation also of the native sBL. Peri‐bacterial degradation of the sBL was also confirmed on the native sBL of the JE (Fig. 7). The sBL of enamel organ caps incubated with *A. actinomycetemcomitans* as a negative control exhibited no peri‐bacterial destruction, even after 6 h (Figure S7A). This observation is consistent with the notion that proteolytic activity from *P. gingivalis* is likely to be responsible for the alterations in sBL. Also, while not excluding any contribution by electron shadowing, this control suggests that the effect does not result from an imaging artefact. This conclusion is reinforced by the fact that after 6 h of exposure, the sBL in contact with *P. gingivalis* appears rough and cavitated (Figs 6 and 7).

## Discussion

Distinctively from epithelial–connective tissue interfaces, where typical basal laminae are implicated, the JE must adhere to mineralized surfaces. To achieve this, it produces an adhesive extracellular matrix composed of four epithelial proteins that form the supramolecular network of an sBL 29. Our work aimed to determine the direct impact of selected periodontopathogenic bacteria on the integrity of the sBL and its constituent proteins. Bacterial biofilms in the gingival sulcus trigger inflammatory processes 8. Once inflammation occurs, it is impossible to dissociate the tissue‐destructive effects of bacteria from those caused by inflammatory cells. Previous studies on bacterially released enzymes and products have largely focused on the destruction of connective tissue components that lie below the JE 17. This study innovates by its focus on the sBL in a context in which inflammation does not intervene. In addition to in vitro assays, we reconstituted an sBL‐like structure 5 and used enamel organ and JE tissue samples 20 to perform ex vivo studies on the native sBL itself. We screened several species of periodontopathogenic bacteria in protein degradation assays. For analyzing sBL degradation, we focused on *P. gingivalis* as it is a major contributor to periodontal diseases 30. The results of in vitro and ex vivo experiments, presented here, demonstrate that some periodontopathogenic bacteria can degrade sBL proteins and affect the structural integrity of both reconstituted and native sBLs. *Porphyromonas gingivalis* has also been found to disseminate systemically, and clearly any alteration of sBL would favor such propagation (31).

When the equilibrium of the oral microbiome is compromised, the numbers of virulent late colonizers, such as *P. gingivalis* and *T. denticola*, are increased in the oral biofilm. This is usually preceded by bridging colonizers that prime the environment to facilitate the propagation of bacteria 12. *Prevotella intermedia*,* F. nucleatum*, and *A. actinomycetemcomitans* are part of this latter cluster. We found that *P. intermedia* exhibited potential similar to that of *P. gingivalis* and *T. denticola* to cleave sBL proteins, but that *F. nucleatum* and *A. actinomycetemcomitans* do not cleave sBL proteins. These data suggest that degradation of the sBL is not exclusive to late colonizers and are coherent with studies reporting a low presence of *A. actinomycetemcomitans* in patients with periodontal diseases 30, 32. Therefore, *P. intermedia,* a member of the bridging colonizers, could technically act to ‘weaken’ the gingival seal, thereby facilitating infiltration of the more aggressive, late‐colonizing species into the gingiva. Continuous adulteration of the sBL by bacterial enzymes would slow down any effort of the JE to reattach to the tooth mineral. This may lead to a self‐perpetuating cycle whereby, as the pocket increases in size, there will be more space for bacterial colonization and a larger surface area for attack of the sBL by proteases, thus reducing JE cell cohesion 15.

*Porphyromonas gingivalis* release a cocktail of enzymes through OMVs or as soluble proteins 33; these enzymes consist of trypsin‐like proteases, such as Arg‐gingipain and Lys‐gingipain 34. The fact that *A. actinomycetemcomitans* (a bacterium which does not produce gingipains) and inactivated *P. gingivalis* have no effect on sBL proteins supports the concept that the proteolytic activity of *P. gingivalis* is responsible for the observed structural alteration of the supramolecular network. Following exposure of AMTN, ODAM, and SCPPPQ1 to *P. gingivalis*, OMVs were observed on the support used for FE‐SEM imaging, and vesicles similar to OMVs 35 were observed on both the reconstituted and the native sBL following exposure to *P. gingivalis*. The translocation of gingipains to OMVs occurs via a recently discovered type IX secretion system (T9SS) 36. Targeting this secretion pathway 37 may hinder the destructive effect we observed on the sBL.

Our findings demonstrate that trypsin‐like proteases efficiently degrade AMTN and ODAM. As such, in vivo proteases from this family could alter the sBL, affecting adhesiveness and ultimately cause JE detachment. Numerous studies have also shown the high sensitivity of Lm332 to trypsin‐like proteases 38. Surprisingly, SCPPPQ1 was not affected by trypsin‐like proteases, which is consistent with its relative resistance to the oral bacteria investigated in this study, possibly resulting from the low number of potential cleavage sites compared with AMTN and ODAM. There are no potential Lys‐gingipains cleavage sites on SCPPPQ1 and only one such site for Arg‐gingipains; however, no degradation was observed despite the fact that together these gingipains act synergistically to increase the trypsin‐like activity of *P. gingivalis* 39. This suggests that protein folding and/or homotypic interactions render this site unavailable for proteolytic degradation. As visualized by FE‐SEM, some fragments of the reconstituted sBL remain on the HOPG surface following exposure to bacteria. This could reflect a gradual destruction of the sBL network, which eventually could liberate any undigested component**.** The fact that the residual material appears as linear profiles similar to those shown by SCPPPQ1 on HOPG 5, and consistent with its resistance to *P. gingivalis*, suggests that the residual profiles could be SCPPPQ1.

Tryptic digestions of the proteins result in a major fragment of 14 kDa for AMTN and of several low‐molecular‐weight fragments for ODAM. These fragments were also detected by MS following exposure of AMTN and ODAM to *P. gingivalis*. Expression of ODAM is reduced in animal models of periodontal disease induced using *P. gingivalis* 27. Also, in patients with periodontitis, the level of ODAM increases in the fluid (i.e. the crevicular fluid) found in the space between the gingiva and the tooth above the JE. Similarly to ODAM, numerous fragments of Lm332 are also found in the crevicular fluid of patients suffering from periodontal diseases 40. Immunolabeling for AMTN in the JE has been reported to decrease over time in *P. gingivalis*‐infected mice 41. These outcomes are consistent with their high degradative susceptibility to enzymes and bacteria observed in the present study. Studies with gingipain mutant strains could help address the questions of whether both gingipains are needed to degrade the sBL components and whether either has a predominant role. It should also be considered that *P. gingivalis* releases enzymes other than gingipains that could synergistically contribute to degradation of the sBL.

Our work focuses on whether the adhesive extracellular matrix produced by the JE is sensitive to periodontopathogenic bacteria. We show here that enzymes and periodontal pathogens, usually associated with destruction of connective tissue components, can also directly attack the epithelial components constituting the sBL as well as alter the supramolecular organization of this critical adhesive extracellular matrix. This observation means that the sBL is also likely to be a bacterial target during periodontal diseases. Alteration and detachment of the sBL would cause a breach in the epithelial seal around teeth, adding a new structural dimension to the progression of periodontal diseases. A better understanding of the action of bacteria on the sBL may identify therapeutic targets and provide novel strategies to prevent JE detachment very early on in order to prevent initiation and propagation of periodontal pockets 17 and thereby control periodontal disease progression.

#### Conflicts of interest

The authors declare no conflicts of interests.

## Supporting information


**Figure S1.** Western blot analysis of AMTN, ODAM and SCPPPQ1 following exposure to bacteria.
**Figure S2.** SDS‐PAGE and western‐blot analysis of in vitro digestion assays of AMTN and ODAM by *P. gingivalis*.
**Figure S3.** SDS‐PAGE analysis of the in vitro digestion of AMTN and ODAM by *P. gingivalis* in the presence of a cocktail of protease inhibitors.
**Figure S4.** Evaluation of the in vitro digestion of Lm332 over time by *P. gingivalis*.
**Figure S5.** Qualitative evaluation of the bacteria after exposure with ODAM or the buffer.
**Figure S6.** Evaluation of the thickness of the peribacterial destruction of the reconstituted sBL by *P. gingivalis*.
**Figure S7.** Characterization of both reconstituted and native sBLs following exposure to *A. actinomycetemcomitans*.
**Table S1.** Top down Mass Spectrometry of the AMTN fragments created by *P. gingivalis* after 2 h of incubation.
**Table S2.** Top down Mass Spectrometry of the ODAM fragments created by *P. gingivalis* after 2 h of incubation.
**Table S3.** In silico analysis of the cleavage of sBL proteins.
