# Supplementary material for: Selective bacterial degradation of the extracellular matrix attaching the gingiva to the tooth
Source: Eur J Oral Sci. 2019 Jun 22;127(4):313–22. doi: 10.1111/eos.12623 (PMC6771947; doi:10.1111/eos.12623)
Supplement: Supplementary file 1 — Figure S1. Western blot analysis of AMTN, ODAM and SCPPPQ1 following exposure to bacteria. Figure S2. SDS‐PAGE and western‐blot analysis of in vitro digestion assays of AMTN and ODAM by P. gingivalis. Figure S3. SDS‐PAGE analysis of the in vitro digestion of AMTN and ODAM by P. gingivalis in the presence of a cocktail of protease inhibitors. Figure S4. Evaluation of the in vitro digestion of Lm332 over time by P. gingivalis. Figure S5. Qualitative evaluation of the bacteria after exposure with ODAM or the buffer. Figure S6. Evaluation of the thickness of the peribacterial destruction of the reconstituted sBL by P. gingivalis. Figure S7. Characterization of both reconstituted and native sBLs following exposure to A. actinomycetemcomitans. Table S1. Top down Mass Spectrometry of the AMTN fragments created by P. gingivalis after 2 h of incubation. Table S2. Top down Mass Spectrometry of the ODAM fragments created by P. gingivalis after 2 h of incubation. Table S3. In silico analysis of the cleavage of sBL proteins. [file EOS-127-313-s001.pdf]

SUPPORTING INFORMATION

Selective bacterial degradation of the extracellular matrix attaching the gingiva to the tooth

AURÉLIEN FOUILLEN, DANIEL GRENIER, JEAN BARBEAU, CHRISTIAN BARON, PIERRE MOFFATT, ANTONIO NANCI

Faculty of Medicine, Université de Montréal, Montréal, Québec, Canada;
Faculty of Dentistry, Université Laval, Quebec City, Québec, Canada;
Shriners Hospital for Children, Montréal, Québec, Canada

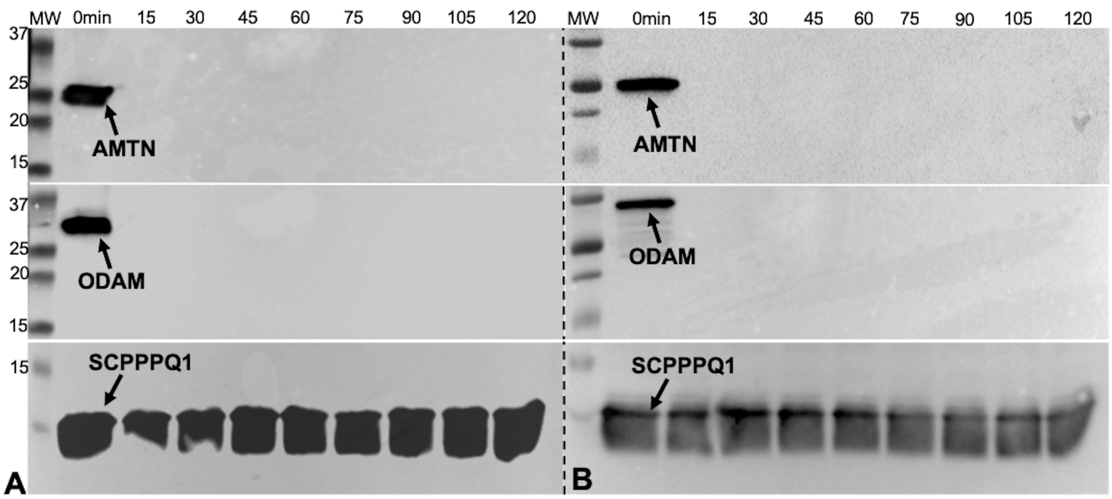

Figure S1: Western blot analysis of AMTN, ODAM and SCPPPQ1 following exposure to bacteria. (**A**) *P. intermedia* and (**B**) *T. denticola* degrade AMTN and ODAM under 15 min, but SCPPPQ1 is not affected. MW in kDa.

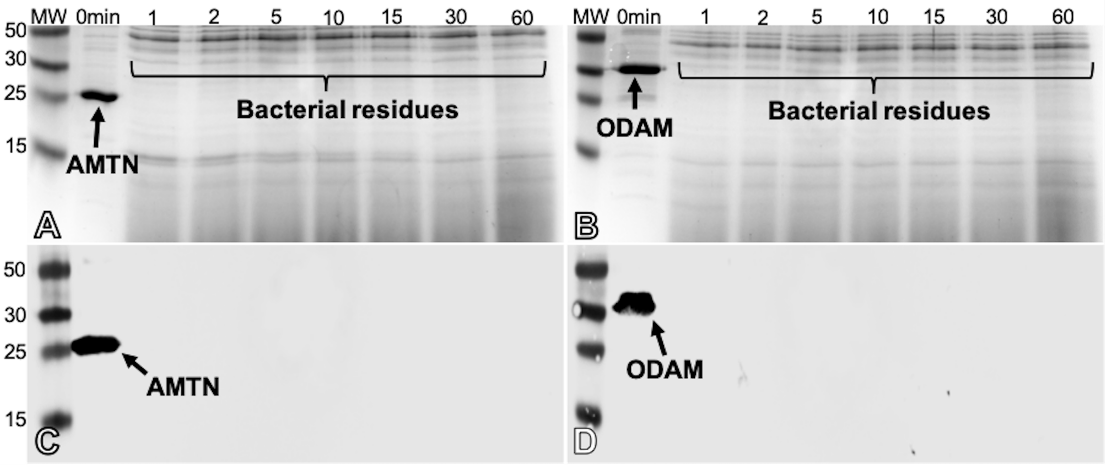

Figure S2: (**A-B**) SDS-PAGE and (**C-D**) western-blot analysis of *in vitro* digestion assays of AMTN and ODAM by *P. gingivalis.* (**A-C**) AMTN (**B-D**) and ODAM are digested totally and extremely rapidly (under 1 min). MW in kDa.

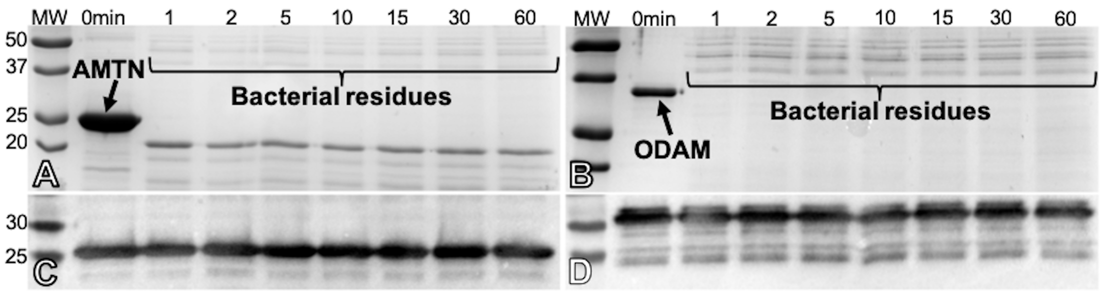

<u>Figure S3</u>: SDS-PAGE analysis of the *in vitro* digestion of (**A**) AMTN (**B**) and ODAM by *P. gingivalis* in the presence of a cocktail of protease inhibitors. While AMTN is still partially digested, ODAM is completely degraded within as little as one minute. Western blot analysis of (**C**) AMTN (**D**) and ODAM after exposition with *P. gingivalis* previously fixed with 2,5% glutaraldehyde show no degradation of the proteins. MW in kDa.

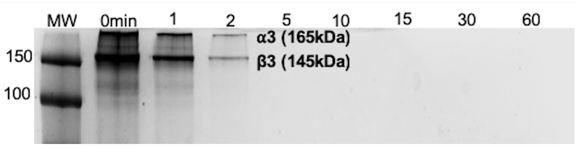

<u>Figure S4</u>: Evaluation of the *in vitro* digestion of Lm332 over time by *P. gingivalis*. The sub-units of Lm332 are susceptible to proteases released by the bacteria under 5 min. MW in kDa.

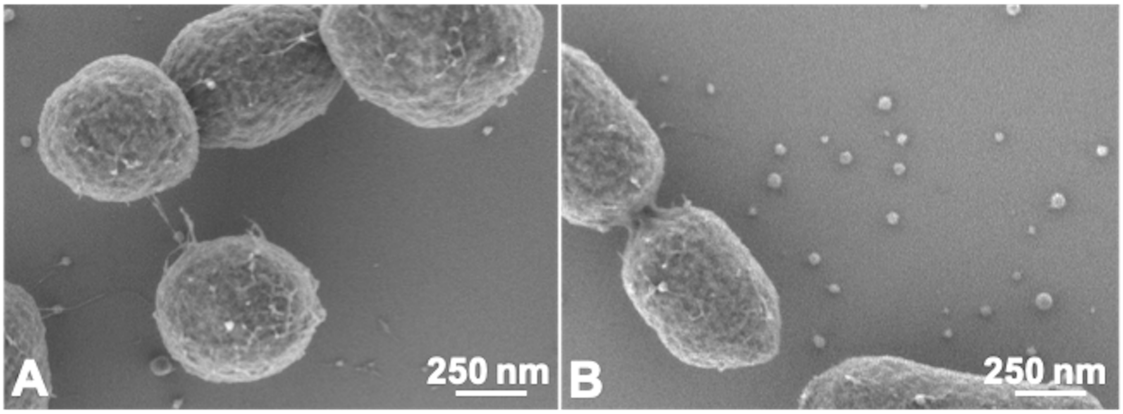

<u>Figure S5</u>: Qualitative evaluation of the bacteria after exposure with ODAM or the buffer. FE-SEM of bacteria after a two hours exposure to (**A**) buffer alone and (**B**) buffer containing ODAM. Following exposure to the protein, more outer membrane vesicles (arrows) appear on the sample surface.

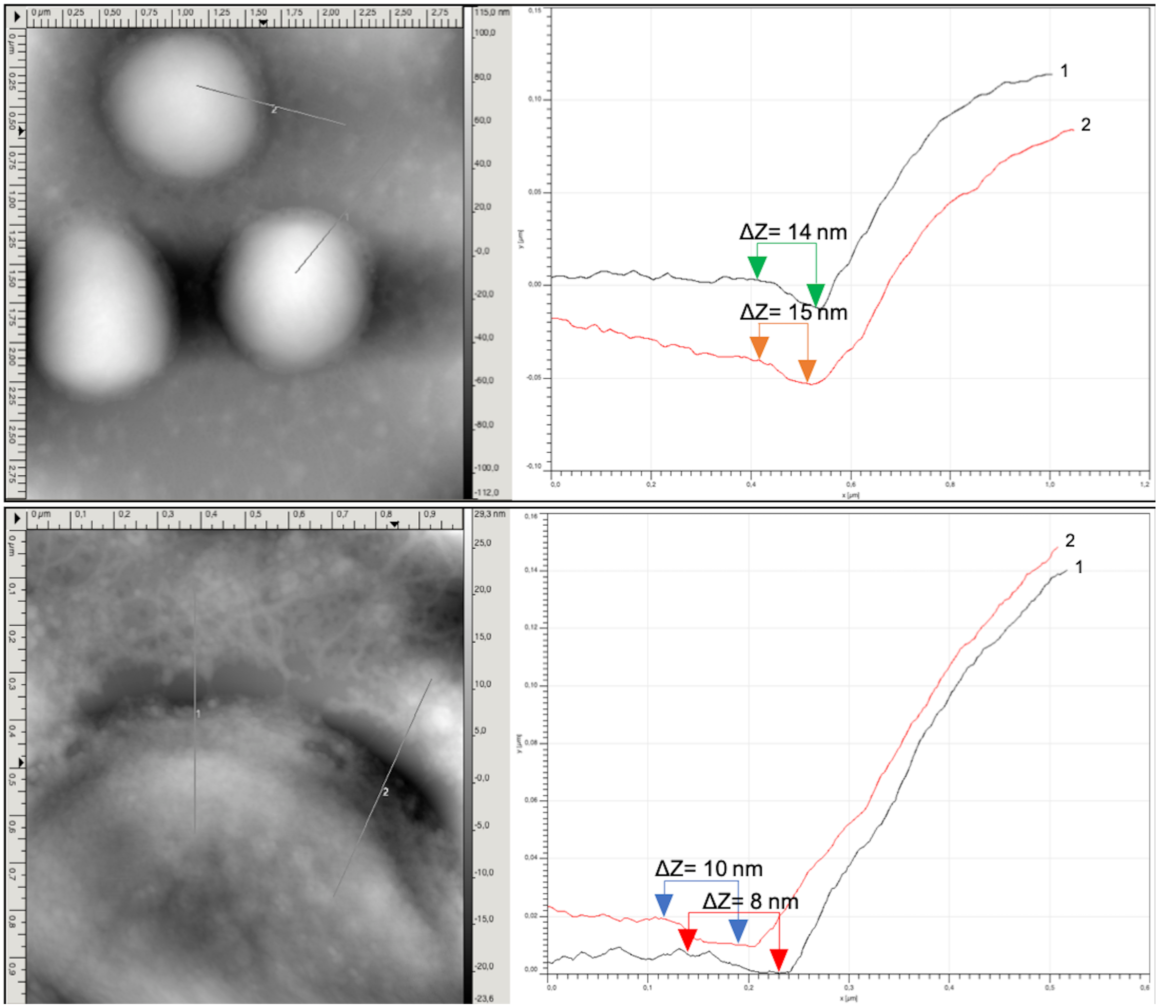

Figure S6: Evaluation of the thickness of the peribacterial destruction of the reconstituted sBL by *P. gingivalis*. AFM analysis shows that the depth of the resulting crater ranges to 8 to 15 nm.

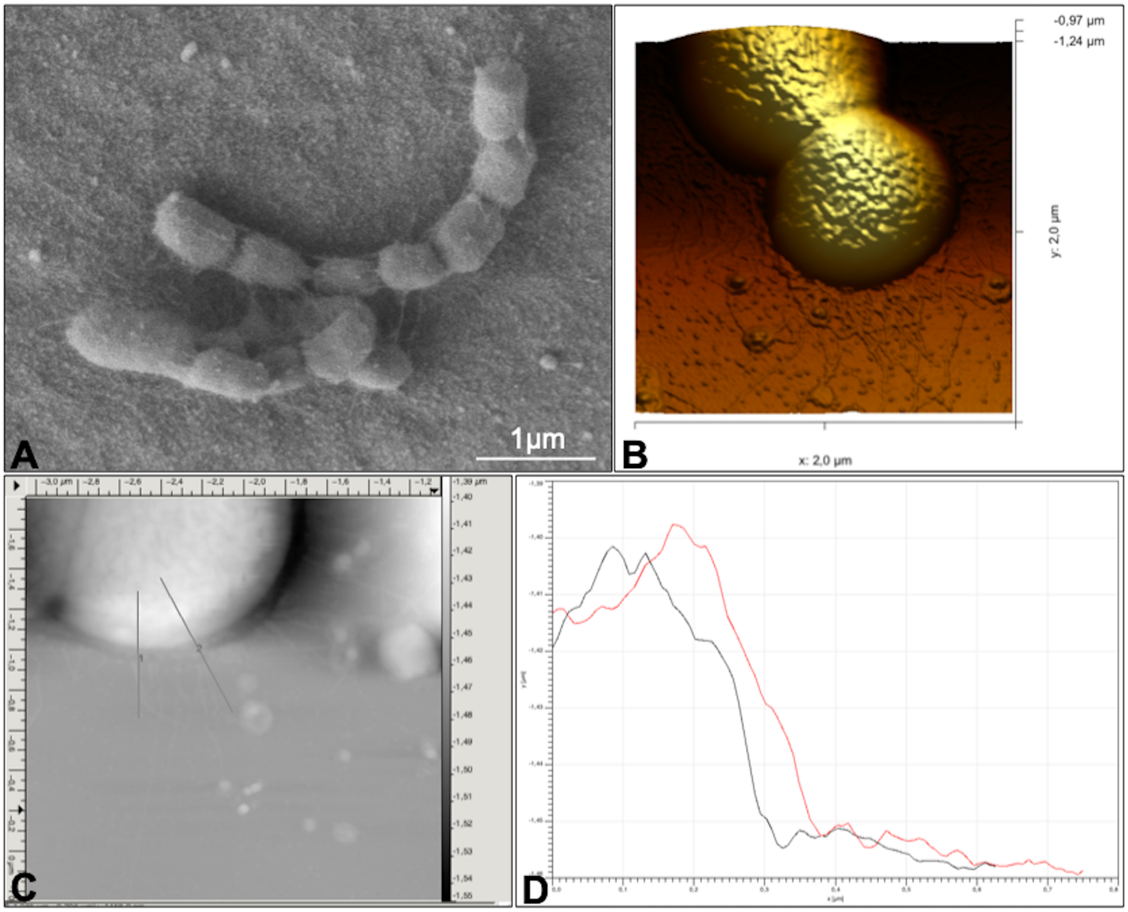

<u>Figure S7</u>: Characterization of both reconstituted and native sBLs following exposure to *A. Actinomycetemcomitans*. (**A**) No alteration of the sBL was observed by FE-SEM imaging of an enamel organ cap exposed for six hours to the bacteria. (**B-C**) AFM visualization of a similary exposed reconstituted sBL shows no peribacterial alterations, (**D**) as confirmed by surface depth measurements.

**<u>Table S1</u>: Top down Mass Spectrometry of the AMTN fragments created by *P. gingivalis* after 2 h of incubation**	
Fragments	***Observed Mass (kDa)***
LAPDQGTLPNQQQSNQVFPSLSLIPLTQMLTLGPDLHLLNPAAGMTPGTQTHPLTLGGLNVQQQLHPHVLPIFVTQLGAQGTILSSEELPQIFTSLIIHSLFPGGILPTSQAGANPDVQDGSLPAGGAGVNPATQGTPAGR	14461,46
GGTRPLQTSGSMLPQLKPALGLPPTKLAPDQGTLPNQQQSNQVFPSLSLIPLTQMLTLGPDLHLLNPAAGMTPGTQTHPLTLGGLNVQQQLHPHVLPIFVTQLGAQGTILSSEELPQIFTSLIIHSLFPGGILPTSQ	14407,47
PLTQMLTLGPDLHLLNPAAGMTPGTQTHPLTLGGLNVQQQLHPHVLPIFVTQLGAQGTILSSEELPQIFTSLIIHSLFPGGILPTSQAGANPDVQDGSLPAGGAGVNPATQGTPAGRLPTPSGTDDD	12891,66
LAPDQGTLPNQQQSNQVFPSLSLIPLTQMLTLGPDLHLLNPAAGMTPGTQTHPLTLGGLNVQQQLHPHVLPIFVTQLGAQGTILSSEELPQIFTSLIIHSLFPGGILPTSQAGANPDVQ	12529,65
PLTQMLTLGPDLHLLNPAAGMTPGTQTHPLTLGGLNVQQQLHPHVLPIFVTQLGAQGTILSSEELPQIFTSLIIHSLFPGGILPTSQAGANPDVQDGSLPAGGAGVNPATQGTPAGRLPTPSGT	12529,57
LAPDQGTLPNQQQSNQVFPSLSLIPLTQMLTLGPDLHLLNPAAGMTPGTQTHPLTLGGLNVQQQLHPHVLPIFVTQLGAQGTILSSEELPQIFTSLIIHSLFPGGILPTSQAGANPDVQ	12529,51
IPLTQMLTLGPDLHLLNPAAGMTPGTQTHPLTLGGLNVQQQLHPHVLPIFVTQLGAQGTILSSEELPQIFTSLIIHSLFPGGILPTSQAGANPDVQDGSLPAGGAGVNPATQGTPAGRL	12113,31
LAPDQGTLPNQQQSNQVFPSLSLIPLTQMLTLGPDLHLLNPAAGMTPGTQTHPLTLGGLNVQQQLHPHVLPIFVTQLGAQGTILSSEELPQIFTSLIIHSLFPGGILPTSQAGAN	12091,40
LLNPAAGMTPGTQTHPLTLGGLNVQQQLHPHVLPIFVTQLGAQGTILSSEELPQIFTSLIIHSLFPGGILPTSQAGANPDVQDGSLPAGGAGVNPATQGTPAGRLPTPSGTDDD	11469,95
MLTLGPDLHLLNPAAGMTPGTQTHPLTLGGLNVQQQLHPHVLPIFVTQLGAQGTILSSEELPQIFTSLIIHSLFPGGILPTSQAGANPDVQDGSLPAGGAGVNPATQGTPAGR	11446,96
TLGPDLHLLNPAAGMTPGTQTHPLTLGGLNVQQQLHPHVLPIFVTQLGAQGTILSSEELPQIFTSLIIHSLFPGGILPTSQAGANPDVQDGSLPAGGAGVNPATQGTPAGR	11201,83
MGSSHHHHHHENLYFQGGTMLGQSGGSSSEQRFNLYPPQILPFFPQFPLPQAPLIPIPFPFPFDPNQVLTPNQLLALITSILNQLQGFLGR	11138,90
SNQVFPSLSLIPLTQMLTLGPDLHLLNPAAGMTPGTQTHPLTLGGLNVQQQLHPHVLPIFVTQLGAQGTILSSEELPQIFTSLIIHSLFPGGILPTSQAGANPDVQ	11138,89
GPDLHLLNPAAGMTPGTQTHPLTLGGLNVQQQLHPHVLPIFVTQLGAQGTILSSEELPQIFTSLIIHSLFPGGILPTSQAGANPDVQDGSLPAGGAGVNPATQGTPAGR	10988,68
QVFPSLSLIPLTQMLTLGPDLHLLNPAAGMTPGTQTHPLTLGGLNVQQQLHPHVLPIFVTQLGAQGTILSSEELPQIFTSLIIHSLFPGGILPTSQAGANPDVQ	10937,81
MLTLGPDLHLLNPAAGMTPGTQTHPLTLGGLNVQQQLHPHVLPIFVTQLGAQGTILSSEELPQIFTSLIIHSLFPGGILPTSQAGANPDVQDGSLPAGGAGVN	10509,48
LLNPAAGMTPGTQTHPLTLGGLNVQQQLHPHVLPIFVTQLGAQGTILSSEELPQIFTSLIIHSLFPGGILPTSQAGANPDVQDGSLPAGGAGVNPATQGTPAGR	10469,44
GSSHHHHHHENLYFQGGTMLGQSGGSSSEQRFNLYPPQILPFFPQFPLPQAPLIPIPFPFPFDPNQVLTPNQLLALITSILNQLQGFLGR	10469,44
LLNPAAGMTPGTQTHPLTLGGLNVQQQLHPHVLPIFVTQLGAQGTILSSEELPQIFTSLIIHSLFPGGILPTSQAGANPDVQDGSLPAGGAGVNPATQGTPAGR	10469,44

SNQVFPSLSLIPLTQMLTLGPDLHLLNPAAGMTPGTQTHPLTLGGLNVQQQLHP HVLPIFVTQLGAQGTILSSEELPQIFTSLIIHSLFPGGILPTSQ	10386,54
LAPDQGTLPNQQQSNQVFPSLSLIPLTQMLTLGPDLHLLNPAAGMTPGTQTHPL TLGGLNVQQQLHPHVLPIFVTQLGAQGTILSSEELPQIF	9915,24
GSSHHHHHHENLYFQGGTMLGQSGGSSSEQRFNLYPPQILPFFPQFPLPQAPL IPIPFPFPFDPNQVLTPNQLLALITSILNQLQGFLGR	9514,03
MLTLGPDLHLLNPAAGMTPGTQTHPLTLGGLNVQQQLHPHVLPIFVTQLGAQGT ILSSEELPQIFTSLIIHSLFPGGILPTSQAGANPDVQ	9514,03
MGSSHHHHHHENLYFQGGTMLGQSGGSSSEQRFNLYPPQILPFFPQFPLPQA PLIPIPFPFPFDPNQVLTPNQLLALITSILNQLQGFLGR	9514,02
MLTLGPDLHLLNPAAGMTPGTQTHPLTLGGLNVQQQLHPHVLPIFVTQLGAQGT ILSSEELPQIFTSLIIHSLFPGGILPTSQAGANPDVQ	9514,02
GSSHHHHHHENLYFQGGTMLGQSGGSSSEQRFNLYPPQILPFFPQFPLPQAPL IPIPFPFPFDPNQVLTPNQLLALITSILNQLQGFLGR	9514,02
MLTLGPDLHLLNPAAGMTPGTQTHPLTLGGLNVQQQLHPHVLPIFVTQLGAQGT ILSSEELPQIFTSLIIHSLFPGGILPTSQAGANPDVQ	9514,02
THPLTLGGLNVQQQLHPHVLPIFVTQLGAQGTILSSEELPQIFTSLIIHSLFPGGIL PTSQAGANPDVQDGSLPAGGAGVNPATQGTPAGR	9216,83
GSSHHHHHHENLYFQGGTMLGQSGGSSSEQRFNLYPPQILPFFPQFPLPQAPL IPIPFPFPFDPNQVLTPNQLLALITSILNQLQGFLGR	9074,85
MLTLGPDLHLLNPAAGMTPGTQTHPLTLGGLNVQQQLHPHVLPIFVTQLGAQGT ILSSEELPQIFTSLIIHSLFPGGILPTSQAGAN	9074,85
TLGGLNVQQQLHPHVLPIFVTQLGAQGTILSSEELPQIFTSLIIHSLFPGGILPTSQ AGANPDVQDGSLPAGGAGVNPATQGTPAGR	8769,57
MLTLGPDLHLLNPAAGMTPGTQTHPLTLGGLNVQQQLHPHVLPIFVTQLGAQGT ILSSEELPQIFTSLIIHSLFPGGILPTSQ	8761,66
LLNPAAGMTPGTQTHPLTLGGLNVQQQLHPHVLPIFVTQLGAQGTILSSEELPQI FTSLIIHSLFPGGILPTSQAGANPDVQ	8536,52
SNQVFPSLSLIPLTQMLTLGPDLHLLNPAAGMTPGTQTHPLTLGGLNVQQQLHP HVLPIFVTQLGAQGTILSSEELPQ	8265,33
VQQQLHPHVLPIFVTQLGAQGTILSSEELPQIFTSLIIHSLFPGGILPTSQAGANPD VQDGSLPAGGAGVNPATQGTPAGR	8214,26
QQLHPHVLPIFVTQLGAQGTILSSEELPQIFTSLIIHSLFPGGILPTSQAGANPDVQ DGSLPAGGAGVNPATQGTPAGR	7987,16
MLTLGPDLHLLNPAAGMTPGTQTHPLTLGGLNVQQQLHPHVLPIFVTQLGAQGT ILSSEELPQIFTSLIIH	7564,02
TQTHPLTLGGLNVQQQLHPHVLPIFVTQLGAQGTILSSEELPQIFTSLIIHSLFPG GILPTSQAGANPDVQ	7514,01
VLPIFVTQLGAQGTILSSEELPQIFTSLIIHSLFPGGILPTSQAGANPDVQDGSLPA GGAGVNPATQGTPAGR	7246,76
LAPDQGTLPNQQQSNQVFPSLSLIPLTQMLTLGPDLHLLNPAAGMTPGTQTHPL TLGGLNVQQQLHPH	7234,72
MLTLGPDLHLLNPAAGMTPGTQTHPLTLGGLNVQQQLHPHVLPIFVTQLGAQGT ILSSEELPQIF	6899,64
THPLTLGGLNVQQQLHPHVLPIFVTQLGAQGTILSSEELPQIFTSLIIHSLFPGGIL PTSQAGAN	6845,68
TLGPDLHLLNPAAGMTPGTQTHPLTLGGLNVQQQLHPHVLPIFVTQLGAQGTIL SSEELPQIF	6655,52
MLTLGPDLHLLNPAAGMTPGTQTHPLTLGGLNVQQQLHPHVLPIFVTQLGAQGT ILSSEELPQ	6639,49
LAPDQGTLPNQQQSNQVFPSLSLIPLTQMLTLGPDLHLLNPAAGMTPGTQTHPL TLGGLNVQ	6493,37
QQLHPHVLPIFVTQLGAQGTILSSEELPQIFTSLIIHSLFPGGILPTSQAGANPDVQ	6055,22

LLNPAAGMTPGTQTHPLTLGGLNVQQQLHPHVLPIFVTQLGAQGTILSSEELPQIF	5922,15
SNQVFPSLSLIPLTQMLTLGPDLHLLNPAAGMTPGTQTHPLTLGGLNVQQQLHPH	5843,05
LAPDQGTLPNQQQSNQVFPSLSLIPLTQMLTLGPDLHLLNPAAGMTPGTQTHPL	5711,93
LLNPAAGMTPGTQTHPLTLGGLNVQQQLHPHVLPIFVTQLGAQGTILSSEELPQ	5661,99
SSEELPQIFTSLIIHSLFPGGILPTSQAGANPDVQDGSLPAGGAGVNPATQGTPAGR	5595,81
VLPIFVTQLGAQGTILSSEELPQIFTSLIIHSLFPGGILPTSQAGANPDVQ	5313,85
LAPDQGTLPNQQQSNQVFPSLSLIPLTQMLTLGPDLHLLNPAAGMTPGTQ	5266,69
IFTSLIIHSLFPGGILPTSQAGANPDVQDGSLPAGGAGVNPATQGTPAGR	4824,48
MLTLGPDLHLLNPAAGMTPGTQTHPLTLGGLNVQQQLHPHVLPIF	4787,54
THPLTLGGLNVQQQLHPHVLPIFVTQLGAQGTILSSEELPQIFT	4771,57
TSLIIHSLFPGGILPTSQAGANPDVQDGSLPAGGAGVNPATQGTPAGR	4565,31
VLPIFVTQLGAQGTILSSEELPQIFTSLIIHSLFPGGILPTSQ	4561,50
SLIIHSLFPGGILPTSQAGANPDVQDGSLPAGGAGVNPATQGTPAGR	4464,26
THPLTLGGLNVQQQLHPHVLPIFVTQLGAQGTILSSEELPQ	4410,37
IIHSLFPGGILPTSQAGANPDVQDGSLPAGGAGVNPATQGTPAGR	4264,15
TLGGLNVQQQLHPHVLPIFVTQLGAQGTILSSEELPQIF	4222,28
QQLHPHVLPIFVTQLGAQGTILSSEELPQIFTSLIIH	4104,24
DGSLPAGGAGVNPATQGTPAGRLPTPSGTDDDFAVTTPAGIQR	4090,98
HSLFPGGILPTSQAGANPDVQDGSLPAGGAGVNPATQGTPAGR	4037,98
TSLIIHSLFPGGILPTSQAGANPDVQDGSLPAGGAGVNPATQ	4025,04
LAPDQGTLPNQQQSNQVFPSLSLIPLTQMLTLGPDLH	4012,06
LPTPSGTDDDFAVTTPAGIQRSTHAIEEATTESANGIQ	3897,83
IFTSLIIHSLFPGGILPTSQAGANPDVQDGSLPAGGAGVN	3888,01
LGPDLHLLNPAAGMTPGTQTHPLTLGGLNVQQQLHPH	3873,01
SNQVFPSLSLIPLTQMLTLGPDLHLLNPAAGMTPGTQ	3872,02
TQMLTLGPDLHLLNPAAGMTPGTQTHPLTLGGLNVQQ	3834,97
LFPGGILPTSQAGANPDVQDGSLPAGGAGVNPATQGTPAGR	3813,89
GPDLHLLNPAAGMTPGTQTHPLTLGGLNVQQQLHPH	3759,92
GGLNVQQQLHPHVLPIFVTQLGAQGTILSSEELPQ	3747,99
MLTLGPDLHLLNPAAGMTPGTQTHPLTLGGLNVQQQ	3733,92
TQMLTLGPDLHLLNPAAGMTPGTQTHPLTLGGLNVQ	3706,91
FPGGILPTSQAGANPDVQDGSLPAGGAGVNPATQGTPAGR	3700,80
VQQQLHPHVLPIFVTQLGAQGTILSSEELPQIF	3666,97
TSLIIHSLFPGGILPTSQAGANPDVQDGSLPAGGAGVN	3627,85
PQIFTSLIIHSLFPGGILPTSQAGANPDVQDGSLP	3588,83
SEELPQIFTSLIIHSLFPGGILPTSQAGANPDVQ	3575,85
QQLHPHVLPIFVTQLGAQGTILSSEELPQIFT	3540,90
NSLKRDVSLGGCSLNKPPFLMLLKGSTRFNKT	3521,85

MTPGTQTHPLTLGGLNVQQQLHPHVLPIFVTQ	3501,85
TLGPDLHLLNPAAGMTPGTQTHPLTLGGLNVQQQ	3489,80
MLTLGPDLHLLNPAAGMTPGTQTHPLTLGGLNVQ	3477,81
QQLHPHVLPIFVTQLGAQGTILSSEELPQIF	3439,85
VQQQLHPHVLPIFVTQLGAQGTILSSEELPQ	3406,82
LGPDLHLLNPAAGMTPGTQTHPLTLGGLNVQQQ	3388,76
VLPIFVTQLGAQGTILSSEELPQIFTSLIIH	3363,86
LTLGPDLHLLNPAAGMTPGTQTHPLTLGGLNVQ	3346,77
QLHPHVLPIFVTQLGAQGTILSSEELPQIF	3311,79
LPNVDLVLSQTKQDIARARRLQAEAEEAR	3292,74
LAPDQGTLPNQQQSNQVFPSLSLIPLTQML	3278,70
LLNPAAGMTPGTQTHPLTLGGLNVQQQLHPH	3240,68
TLGPDLHLLNPAAGMTPGTQTHPLTLGGLNVQ	3233,68
LHPHVLPIFVTQLGAQGTILSSEELPQIF	3183,73
QQLHPHVLPIFVTQLGAQGTILSSEELPQ	3179,70
LGPDLHLLNPAAGMTPGTQTHPLTLGGLNVQ	3132,64
LPIFVTQLGAQGTILSSEELPQIFTSLII	3125,44
VLPIFVTQLGAQGTILSSEELPQIFTSLI	3115,47
LTPTFNKLCGNSRQMACTPISCPGELCPQ	3109,47
QLHPHVLPIFVTQLGAQGTILSSEELPQ	3051,64
LHPHVLPIFVTQLGAQGTILSSEELPQI	3034,61
LAPDQGTLPNQQQSNQVFPSLSLIPLTQ	3033,58
IFTSLIIHSLFPGGILPTSQAGANPDVQD	3007,56
THPLTLGGLNVQQQLHPHVLPIFVTQ	2886,58
VLPIFVTQLGAQGTILSSEELPQIFT	2800,52
GPDLHLLNPAAGMTPGTQTHPLTLGGLN	2790,35
HLLNPAAGMTPGTQTHPLTLGGLNVQQ	2765,43
FPGGILPTSQAGANPDVQDGSLPAGGAGVN	2765,43
LLNPAAGMTPGTQTHPLTLGGLNVQQQ	2756,43
VLPIFVTQLGAQGTILSSEELPQIF	2699,48
HLLNPAAGMTPGTQTHPLTLGGLNVQ	2637,37
TSLIIHSLFPGGILPTSQAGANPDVQ	2632,38
PTSQAGANPDVQDGSLPAGGAGVNPATQG	2631,34
QLGAQGTILSSEELPQIFTSLIIH	2594,39
LGPDLHLLNPAAGMTPGTQTHPLTL	2564,34
THPLTLGGLNVQQQLHPHVLPIF	2558,41
LGAQGTILSSEELPQIFTSLIIH	2466,34
LGAQGTILSSEELPQIFTSLIIH	2466,33
TLGPDLHLLNPAAGMTPGTQTHPL	2451,26

VLPIFVTQLGAQGTILSSEELPQ	2439,32
LPIFVTQLGAQGTILSSEELPQ	2340,26
IIHSLFPGGILPTSQAGANPDVQ	2331,22
HLLNPAAGMTPGTQTHPLTLGGL	2296,20
PLQTSGSMLPQLKPALGLPPTK	2273,28
MLTLGPDLHLLNPAAGMTPGTQ	2247,14
GPDLHLLNPAAGMTPGTQTHPL	2237,13
PLQTSGSMLPQLKPALGLPPT	2145,19
HLLNPAAGMTPGTQTHPLTL	2069,07
TQLGAQGTILSSEELPQIF	2031,05
TSLIIHSLFPGGILPTSQAG	2008,10
SLFPGGILPTSQAGANPDVQ	1967,99
LLNPAAGMTPGTQTHPLTL	1932,01
LFPGGILPTSQAGANPDVQ	1880,96
LTQMLTLGPDLHLLNPA	1845,99
SNQVFPSLSLIPLTQM	1773,93
GSMLPQLKPALGLPPTK	1747,00
PLQTSGSMLPQLKPAL	1679,93
VLPIFVTQLGAQGTIL	1668,98
HSLFPGGILPTSQAGAN	1665,85
QQLHPHVLPIFVTQ	1655,91
QSNQVFPSLSLIPLT	1642,89
SNQVFPSLSLIPLT	1514,83
IIHSLFPGGILPTS	1450,82
LVGGPTAPGSGHTI	1262,67
IFTSLIIHS	1029,59
SLIPLTQML	1014,58
IFTSLIIH	942,55

<u>Table S1</u>: Top down Mass Spectrometry of the AMTN fragments created by P. gingivalis after 2 h of incubation.

**<u>Table S2</u> : Top down Mass Spectrometry of the ODAM fragments created by *P. gingivalis* after 2 h of incubation**	
Fragments	***Observed Mass (kDa)***
MLTLGPDLHLLNPAAGMTPGTQTHPLTLGGLNVQQQLHPHVLPIFVTQLGAQGT ILSSEELPQIFTSLIIHSLFPGGILPTSQAGANPDVQDGSLPAGGAGVNPATQGTP AGR	11446,98
EQPQQTVPRSPQQTRQQQYEEQIPFYAQFGYIPQLAEPAISGGQQQLAFDPQL GTAPEIAVMSTGEEIPYLQK	8214,12
QQQYEEQIPFYAQFGYIPQLAEPAISGGQQQLAFDPQLGTAPEIAVMSTGEEIPY LQ	6327,05
ALDQFAGLLPNQIPLTGEASFAQGAQAGQVDPLQLQTPPQTQPGPSHVMPYVF SFK	5931,98
DQFAGLLPNQIPLTGEASFAQGAQAGQVDPLQLQTPPQTQPGPSHVMPYVFSF K	5747,87
QQQYEEQIPFYAQFGYIPQLAEPAISGGQQQLAFDPQLGTAPEIAVMS	5296,56
GLLPNQIPLTGEASFAQGAQAGQVDPLQLQTPPQTQPGPSHVMPYVFSFK	5286,67
LLPNQIPLTGEASFAQGAQAGQVDPLQLQTPPQTQPGPSHVMPYVFSFK	5229,65
QQQYEEQIPFYAQFGYIPQLAEPAISGGQQQLAFDPQLGTAPEIAVM	5209,53
PQLAEPAISGGQQQLAFDPQLGTAPEIAVMSTGEEIPYLQKEAINFRH	5192,50
YAQFGYIPQLAEPAISGGQQQLAFDPQLGTAPEIAVMSTGEEIPYLQ	5036,47
AQFGYIPQLAEPAISGGQQQLAFDPQLGTAPEIAVMSTGEEIPYLQK	5001,50
QQQYEEQIPFYAQFGYIPQLAEPAISGGQQQLAFDPQLGTAPEIA	4979,43
QYEEQIPFYAQFGYIPQLAEPAISGGQQQLAFDPQLGTAPEIAVM	4953,41
LMSASNSNELLLNLNNGQLLPLQLQGPLNSWIPPFSGILQQQQQ	4857,51
FGYIPQLAEPAISGGQQQLAFDPQLGTAPEIAVMSTGEEIPYLQK	4802,41
QQQYEEQIPFYAQFGYIPQLAEPAISGGQQQLAFDPQLGTAPE	4795,30
PFYAQFGYIPQLAEPAISGGQQQLAFDPQLGTAPEIAVMSTGEEI	4778,28
PLTGEASFAQGAQAGQVDPLQLQTPPQTQPGPSHVMPYVFSFKMP	4775,36
HDSAGVFMPSTSPKPSTTNVFTSAVDQTITPELPEEKDKTDSLR	4761,29
FGYIPQLAEPAISGGQQQLAFDPQLGTAPEIAVMSTGEEIPYLQ	4674,32
IPLTGEASFAQGAQAGQVDPLQLQTPPQTQPGPSHVMPYVFSFK	4664,33
GYIPQLAEPAISGGQQQLAFDPQLGTAPEIAVMSTGEEIPYLQK	4655,34
SASNSNELLLNLNNGQLLPLQLQGPLNSWIPPFSGILQQQQQ	4613,38
LMSASNSNELLLNLNNGQLLPLQLQGPLNSWIPPFSGILQQQ	4601,39
YIPQLAEPAISGGQQQLAFDPQLGTAPEIAVMSTGEEIPYLQK	4598,33
QYEEQIPFYAQFGYIPQLAEPAISGGQQQLAFDPQLGTAPE	4539,19
SASNSNELLLNLNNGQLLPLQLQGPLNSWIPPFSGILQQQQ	4485,32
YISQKVSDASDKTQQAERALGSAAADAQRAKNGAGEALEISSE	4419,21
PLTGEASFAQGAQAGQVDPLQLQTPPQTQPGPSHVMPYVFSF	4419,21
QIPFYAQFGYIPQLAEPAISGGQQQLAFDPQLGTAPEIAVM	4404,22

QQQQAQIPGLSQFSLSALDQFAGLLPNQIPLTGEASFAQGAQ	4397,23
QQQYEEQIPFYAQFGYIPQLAEPAISGGQQQLAFDPQLG	4397,12
PGLSQFSLSALDQFAGLLPNQIPLTGEASFAQGAQAGQVDPLQ	4380,19
LMSASNSNELLLNLNNGQLLPLQLQGPLNSWIPPFSGILQ	4345,27
TGEASFAQGAQAGQVDPLQLQTPPQTQPGPSHVMPYVFSFK	4344,12
QQQYEEQIPFYAQFGYIPQLAEPAISGGQQQLAFDPQ	4227,02
TGEASFAQGAQAGQVDPLQLQTPPQTQPGPSHVMPYVFSF	4213,02
EASFAQGAQAGQVDPLQLQTPPQTQPGPSHVMPYVFSFK	4183,04
QQQQAQIPGLSQFSLSALDQFAGLLPNQIPLTGEASFAQ	4141,11
ALDQFAGLLPNQIPLTGEASFAQGAQAGQVDPLQLQTPPQ	4125,06
LGTAPEIAVMSTGEEIPYLQKEAINFRHDSAGVFMPST	4106,06
AKFLGNQQVSYGQSLSFDYRVDRGGRHPSAHDVILEG	4106,06
LAEPAISGGQQQLAFDPQLGTAPEIAVMSTGEEIPYLQK	4097,06
TGEASFAQGAQAGQVDPLQLQTPPQTQPGPSHVMPYVFS	4065,95
QAQIPGLSQFSLSALDQFAGLLPNQIPLTGEASFAQGAQ	4013,05
AEPAISGGQQQLAFDPQLGTAPEIAVMSTGEEIPYLQK	3983,98
TGEASFAQGAQAGQVDPLQLQTPPQTQPGPSHVMPYVF	3978,92
LAEPAISGGQQQLAFDPQLGTAPEIAVMSTGEEIPYLQ	3968,97
DPVAGDIKGCDCNLEGVLPEICDAHGRCLCRPGVEGPR	3960,89
HDSAGVFMPSTSPKPSTTNVFTSAVDQTITPELPEEK	3944,90
PLQLQGPLNSWIPPFSGILQQQQQAQIPGLSQFSLS	3943,08
SNSNELLLNLNNGQLLPLQLQGPLNSWIPPFSGILQ	3941,08
YAQFGYIPQLAEPAISGGQQQLAFDPQLGTAPEIAVM	3918,95
EPAISGGQQQLAFDPQLGTAPEIAVMSTGEEIPYLQK	3912,94
FAQGAQAGQVDPLQLQTPPQTQPGPSHVMPYVFSFK	3895,93
QQAQIPGLSQFSLSALDQFAGLLPNQIPLTGEASFAQ	3884,99
TGEASFAQGAQAGQVDPLQLQTPPQTQPGPSHVMPYV	3831,85
QAQIPGLSQFSLSALDQFAGLLPNQIPLTGEASFAQ	3756,93
AQFGYIPQLAEPAISGGQQQLAFDPQLGTAPEIAVM	3755,88
AQGAQAGQVDPLQLQTPPQTQPGPSHVMPYVFSFK	3748,86
QQQYEEQIPFYAQFGYIPQLAEPAISGGQQQLA	3739,81
TGEASFAQGAQAGQVDPLQLQTPPQTQPGPSHVMPY	3732,78
SASNSNELLLNLNNGQLLPLQLQGPLNSWIPPFS	3689,90
ISGGQQQLAFDPQLGTAPEIAVMSTGEEIPYLQK	3614,66
MPQEQGQMFQYYPVYMVLPWEQPQQTVPR	3598,67
FGYIPQLAEPAISGGQQQLAFDPQLGTAPEIAVM	3556,79
GAQAGQVDPLQLQTPPQTQPGPSHVMPYVFSFK	3549,77
QQQYEEQIPFYAQFGYIPQLAEPAISGGQQ	3427,63
QAGQVDPLQLQTPPQTQPGPSHVMPYVFSFK	3421,71

GAQAGQVDPLQLQTPPQTQPGPSHVMPYVFSF	3421,67
GGQQQLAFDPQLGTAPEIAVMSTGEEIPYLQK	3415,69
PGLSQFSLSALDQFAGLLPNQIPLTGEASFAQG	3371,74
KSFGLPGDMVLLEKKPDVQLTGQHMSIIYE	3371,74
QEQGQMFQYYPVYMVLPWEQPQQTVPR	3370,58
GQQQLAFDPQLGTAPEIAVMSTGEEIPYLQK	3358,67
QQQQAQIPGLSQFSLSALDQFAGLLPNQIPL	3349,76
LNNGQLLPLQLQGPLNSWIPPFSGILQQQQ	3341,77
TGEASFAQGAQAGQVDPLQLQTPPQTQPGPSHV	3341,59
PSTTNVFTSAVDQTITPELPEEKDKTDSLR	3319,63
PFYAQFGYIPQLAEPAISGGQQQLAFDPQL	3293,68
QGPLNSWIPPFSGILQQQQQAQIPGLSQFS	3293,68
AGQVDPLQLQTPPQTQPGPSHVMPYVFSFK	3293,65
GAQAGQVDPLQLQTPPQTQPGPSHVMPYVFS	3274,60
AGQVDPLQLQTPPQTQPGPSHVMPYVFSF	3165,56
TTNVFTSAVDQTITPELPEEKDKTDSLR	3135,54
QQAQIPGLSQFSLSALDQFAGLLPNQIPL	3093,65
QLAFDPQLGTAPEIAVMSTGEEIPYLQK	3045,54
GSSHHHHHHENLYFQGGTMAPLIPQR	2988,39
GQMFQYYPVYMVLPWEQPQQTVPR	2985,42
PLQLQTPPQTQPGPSHVMPYVFSFK	2823,44
AGQVDPLQLQTPPQTQPGPSHVMPYV	2785,39
PAISGGQQQLAFDPQLGTAPEIAVMSTG	2782,43
PSTTNVFTSAVDQTITPELPEEKDK	2746,35
EPAISGGQQQLAFDPQLGTAPEIAVM	2669,29
QEQGQMFQYYPVYMVLPWEQ	2562,34
PSTTNVFTSAVDQTITPELPEEK	2504,23
SALDQFAGLLPNQIPLTGEASFAQ	2487,26
LQTPPQTQPGPSHVMPYVFSFK	2485,24
LMSASNSNELLLNLNNGQLLPL	2368,23
TTNVFTSAVDQTITPELPEEK	2319,15
TPPQTQPGPSHVMPYVFSFK	2244,10
LQTPPQTQPGPSHVMPYVFS	2210,08
PGLSQFSLSALDQFAGLLPNQ	2204,20
QYEEQIPFYAQFGYIPQ	2119,99
TGEASFAQGAQAGQVDPLQL	1986,96
MPQEQGQMFQYYPVY	1907,82
FGYIPQLAEPAISGGQQQ	1902,95
GILQQQQQAQIPGLSQF	1882,99

TPPQTQPGPSHVMPYVF	1881,91
QQQYEEQIPFYAQFG	1874,85
YMVLPWEQPQQTVPR	1871,92
EEQIPFYAQFGYIPQ	1828,87
LNNGQLLPLQLQGPLN	1731,95
MVLPWEQPQQTVPR	1708,86
TQPGPSHVMPYVFS	1545,73
QQQYEEQIPFYA	1542,70
LDQFAGLLPNQIPL	1537,85
QQQYEEQIPFY	1471,66
EEQIPFYAQF	1270,59
MVLPWEQPQQ	1254,60
WIPPFSGILQ	1156,63
FSLSALDQFAG	1154,56

Table S2: Top down Mass Spectrometry of the ODAM fragments created by P. gingivalis after 2 h of incubation.

**Table S3**: ***In silico*** **analysis of the cleavage of sBL proteins**				
**Proteases**	**Family**	**Cleavage sites**		
		**AMTN**	**ODAM**	**SCPPPQ1**
α-chymotrypsin	Serine proteases	34	56	7
Trypsin	Serine proteases	5	10	2
Elastase	Serine proteases	23	29	9
Subtilisin	non-specific proteases	34	23	6
Endoproteinase Glu-C	Serine proteases	9	11	3
Proteinase K	Serine proteases	99	129	51
Clostripain	Cysteine proteases	4	5	1
Pepsin	Aspartic proteases	47	80	28
Lys-gingipain	Cysteine proteases	2	6	0
Arg-gingipain	Cysteine proteases	4	5	1
Both gingipains	Cysteine proteases	6	11	1

Table S3: ***In silico*** **analysis of the cleavage of sBL proteins.**
